# A method to correct for the influence of bovine serum albumin-associated vitamin D metabolites in protein extracts from neonatal dried blood spots

**DOI:** 10.1186/s13104-022-06077-1

**Published:** 2022-06-03

**Authors:** Sanne Grundvad Boelt, Oleguer Plana-Ripoll, Clara Albiñana, Bjarni Vilhjálmsson, John J. McGrath, Arieh S. Cohen

**Affiliations:** 1 Center for Neonatal Screening, Department of Congenital Disorders—Clinical Mass Spectrometry Statens Serum Institut, Artillerivej 5, DK-2300 Copenhagen S, Denmark; 2 National Centre for Register-Based Research, Department of Economics and Business Economics, Aarhus University, Fuglesangs Allé 26, Building 2640 Aarhus V, DK-8210 Aarhus, Denmark; 3 Department of Clinical Epidemiology, Aarhus University and Aarhus University Hospital, Olof Palmes Allé 43-45 Aarhus N, 8200 Aarhus, Denmark; 4 Bioinformatics Research Centre, Aarhus University, C.F. Møllers Allé 8, Building 1110 Aarhus C, DK-8000 Aarhus, Denmark; 5 Queensland Centre for Mental Health Research, The Park Centre for Mental Health, University of Queensland, St. Lucia, QLD-4072 Australia; 6 Queensland Brain Institute, University of Queensland, St. Lucia, QLD-4072 Australia

**Keywords:** 25 hydroxyvitamin D, Bovine serum albumin, Assay methodology, LC–MS/MS

## Abstract

**Objective:**

We developed an assay to measure the concentration of 25 hydroxyvitamin D_2_ and D_3_ in protein extracts derived from stored neonatal dried blood spots. During this study, we postulated that these samples had been contaminated with exogenous vitamin D metabolites because of the addition of bovine serum albumin (BSA) as part of an extraction step undertaken 7 years earlier. The aim of the current study was to develop methods in order to adjust for this contamination.

**Results:**

We identified between-plate variations in 25 hydroxyvitamin D_2_ and D_3_ concentrations which suggested the presence of three different BSA batches. Based on repeat extraction (without the addition of BSA) and testing of 395 samples, we developed models to correct for the exogenous 25 hydroxyvitamin D_2_ and D_3._ The regression models were Diff_25OHD3_ = − 8.2 + 1.8* Diff_25OHD2_ for low contamination, Diff_25OHD3_ = 23.8 + 1.7* Diff_25OHD2_ for middle contamination, and Diff_25OHD3_ = 14.3 + 3.0* Diff_25OHD2_ for high contamination. After these corrections, the three subsamples had comparable distributions within the expected range for both 25 hydroxyvitamin D_2_ and D_3_.

**Supplementary Information:**

The online version contains supplementary material available at 10.1186/s13104-022-06077-1.

## Introduction

Our group has published several epidemiological studies that have involved the measurement of vitamin D status based on stored neonatal dried blood spots (DBS) [1–3]. In contrast to standard clinical tests for vitamin D status (which are based on several millilitres of serum from venous blood), neonatal DBS provide a very small volume of whole blood (one 3.2 mm DBS punch is equivalent to 1.64 µL whole blood). Because these samples are collected for routine neonatal screening, there is often very little material available for subsequent research studies. Fortunately, with advances in tandem mass spectroscopy and refinements in assays methods, it is now possible to reliably and accurately assess vitamin D status based on small samples from DBS [4–6].

We recently commenced a large project that involved the assessment of neonatal vitamin D status in approximately 76,000 Danish neonates. The Lundbeck Foundation Initiative for Integrated Psychiatric Research (iPSYCH) study is a case-cohort sample, with cases selected on the presence of selected mental disorders, and a randomly selected population-based cohort [7]. Seven years prior to our study, the neonatal DBS from the iPSYCH samples were retrieved and processed in order to extract DNA suitable for genotyping [8–10]. The protein extracts kept after the DNA study were available for future approved research. Our group developed a sensitive and robust assay to measure the concentration of 25 hydroxyvitamin D_3_ (25OHD3) and 25 hydroxyvitamin D_2_ (25OHD2) in the left over protein extracts from the iPSYCH sample [11]. During our analysis, we identified that the iPSYCH samples were returning: (a) very high concentrations of 25OHD3 (often over 100 nmol/L), and (b) the presence of appreciable concentrations of 25OHD2 in the majority of samples (while previous studies had identified 25OHD2 in less than 10% of neonatal DBS, [2, 12]). Here we describe our steps to explore the reasons for these unexpected findings, and explore ways to improve the precision of our previously published assay [11].

As part of the investigations into these findings, we noted that bovine serum albumin (BSA) had been included in the DNA extraction process. BSA is not required (nor recommended) for assays related to 25OHD2 and 25OHD3 assays, but has been recommended as an aid to optimize DNA extraction suitable for amplification [9]. To the best of our knowledge, there have been no prior studies that have tested for the presence of 25OHD_2_ and 25OHD_3_ in BSA. However, it is known that cattle produce vitamin D from bright sunshine [13] and are also often given vitamin D supplements in order to reduce post-weaning hypocalcaemia [14]. We speculated that the unexpectedly high concentrations of 25OHD2 and 25OHD3 identified in our assays were the results of BSA-related contamination with exogenous vitamin D metabolites.

We note that other large population-based studies have used modelling strategies to provide corrected 25OHD3 estimates. The National Health and Nutrition Examination Survey (NHANES) issued corrected values to account for changes in assay accuracy over different waves [15, 16]. The UK Biobank study (n ~ 500,000) used modelling to correct values for a range of biomarkers (including the vitamin D metabolites assessed in the current study) after a dilution artefact during an early wash phase of aliquot handling was detected [17].

The aims of this study were to (a) measure if 25OHD2 and 25OHD3 were present in recently-purchased batches of BSA; (b) re-assay 25OHD2 and 25OHD3 in a subset of the iPSYCH neonatal DBS with the identical extraction steps but without BSA, in order to compare contaminated versus uncontaminated in pairwise samples; and (c) based on the findings from the pairwise comparisons, apply a set of corrections to the full iPSYCH sample in order to derive estimates adjusted for the influence of BSA contamination. These methods can guide researchers to reproduce our steps if BSA contamination has influenced related assays.

## Main text

### Material and methods

In the following section we concisely outline (a) the methods we used to determine if BSA contains 25OHD2 and 25OHD3, (b) the selection of a subsample of DBS for repeat testing without the addition of BSA, and (c) based on the paired contaminated versus uncontaminated samples, the modelling steps we used to apply corrections across the entire sample.

We first assayed the concentration of 25OHD2 and 25OHD3 in 11 individuals using our standard (non-BSA exposed) laboratory protocols (full details are provided in the Supplement). For the high-throughput assessment of 25OHD2 and 25OHD3 concentration in the large iPSYCH sample, the protein extracts were prepared and subsequently assayed in 96-well plates (full details of this assay are available in Boelt et al. [11]). We inspected the mean 25OHD2 and 25OHD3 concentration for each of the 1050 plates, when ordered by the sequence of testing routinely used in the iPSYCH sample (i.e. based on date of birth of the neonates). If the results from the entire iPSYCH sample were uniformly increased, it would suggest that contamination was from one batch of BSA across the entire sample. If there were abrupt between-plate changes in mean 25OHD2 and 25OHD3 concentration, it would suggest that contamination was from more than one BSA batch.

In order to quantify the extent of BSA-related contamination with 25OHD2 and 25OHD3, we chose a subsample of the iPSYCH sample for repeat testing using the identical methods for the preparation of the protein extract (and subsequent assay for 25OHD2 and 25OHD3), but without the addition of BSA (henceforth, the uncontaminated results). By comparing the contaminated versus uncontaminated results in these pairs of samples, we deduced the contribution of the BSA-related 25OHD2 and 25OHD3 to the contaminated results. Finally, based on the repeat-tested subsample, we derived statistical models that adjusted for BSA contamination in the entire iPSYCH sample (henceforth, the corrected results).

## Results

### The concentration of 25OHD2 and 25OHD3 in recently purchased BSA batches

Based on routine laboratory assays of 11 individuals, none of the samples had detectable 25OHD2, while 25OHD3 based on full blood concentration ranged from 11.2 to 58.4 nmol/L. We then assessed the contribution of 17 different batches of recently purchased BSA to the extraction buffer used in the preparation of the DBS protein extract. We found that only two BSA batches had appreciable concentrations of 25OHD2 and 25OHD3. The contribution of these two batches to the samples is shown in the Additional file 1: Table. For example, one BSA batch contributed a mean (standard deviation) of 20.9 (3.0) nmol/L of 25OHD2 and 27.7 (3.0) nmol/L of 25OHD3 in addition to the endogenous 25OHD2 and 25OHD3. Of interest, previous studies by our group had found that fewer than 10% of neonatal dried blood spots had detectable concentrations of 25OHD2 [1, 2, 12, 18–21]. Thus, we concluded that the presence of measurable 25OHD2 in our iPSYCH sample could serve as a proxy marker for probable BSA-related contamination.

### The pattern of contaminated 25OHD2 and 25OHD3 concentration across all samples

**Fig. 1:**
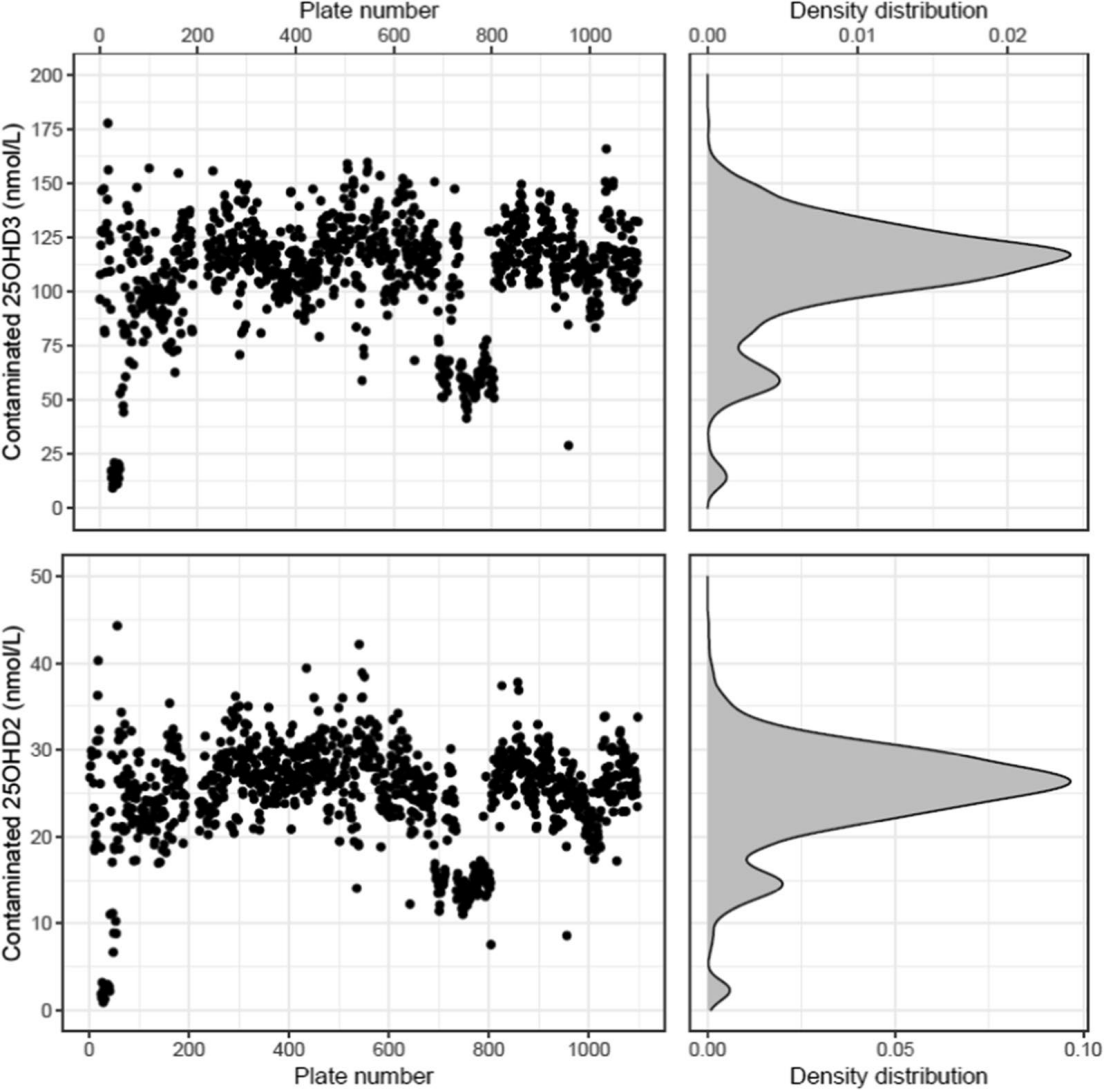
Concentration of mean 25OHD3 (upper panels) and 25OHD2 (lower panels) in nmol/L per plate ordered in the sequence of testing (left sided panels), and density plots to show the distribution of these values (right sided panels)

A total of 76,611 samples were assessed across 1,050 96-well plates. Figure 1 shows the concentration of mean 25OHD2 and 25OHD3 per plate ordered in the sequence of testing (left sided panels), and density plots to show the distribution of these values (right sided panels). Firstly, for the majority of the samples, the concentration of 25OHD3 was generally higher than previous DBS-based studies [18]. Secondly, there was a small but distinct subsample with low (but clinically appropriate) 25OHD3 concentrations (across plate numbers 24–42). Finally, there was a third subsample (across plate numbers 735–804) with middle-range 25OHD3 concentrations. A strikingly similar between-plate pattern was also found for 25OHD2 concentrations. In the absence of documentation of (a) the number of BSA batches that were originally used for DNA extraction, and (b) what concentrations of 25OHD2 and 25OHD3 were in BSA batches used seven years prior to our study, we assumed that there were three different BSA batches used in the prior DNA extraction analyses. We classified all plates into those with low-, middle-, or high-contamination if the contaminated 25OHD2 was ≤ 10, 10–20, or ≥ 20 nmol/L, respectively.

### Comparing paired contaminated versus uncontaminated samples

Based on the three low-, middle- and high-contamination subgroups, we randomly selected 395 individuals for repeat testing. We went back to the original stored DBS to collect samples for protein extraction but without BSA contamination. We used the same protocol for the assessment of 25OHD2 and 25OHD3 as for the full iPSYCH sample [11]. Within this repeat-test sample, two participants with very high concentrations were excluded (25OHD2 = 267 nmol/L; 25OHD3 = 501 nmol/L).

**Fig. 2:**
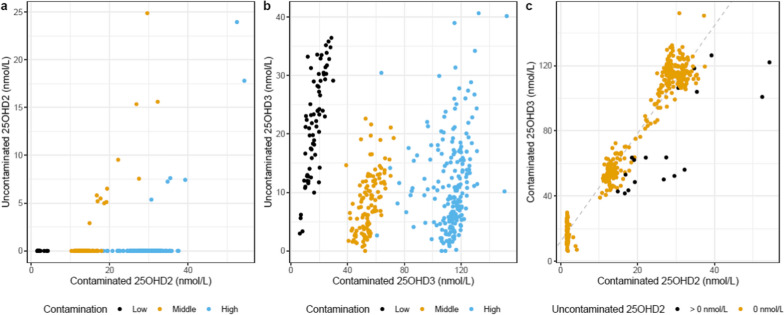
Scatter plots for contaminated versus uncontaminated 25OHD2 (**a**) and 25OHD3 (**b**) in nmol/L in the entire sample according to low-, middle-, and high-contamination of the samples (with average 25OHD2 levels per batch of less than 10 nmol/L, 10–20 nmol/L, or greater than 20 nmol/L, respectively). **c** shows a scatter plot of contaminated 25OHD2 versus contaminated 25OHD3 in nmol/L (with a dashed regression line) according to uncontaminated 25OHD2 being zero or non-zero

Consistent with our previous, independent studies based on Danish neonatal dried blood spots which found 25OHD2 in a minority of the samples, only 18 of the 393 (4.6%) samples had non-zero 25OHD2 concentrations (Fig. 2a). We plotted the contaminated versus uncontaminated measures of 25OHD3 according to the low-, middle- and high-contamination subgroups. There was a clear correlation between these values in each of the subgroups (Fig. 2b). We found a linear relationship between contaminated 25OHD2 and 25OHD3 values (25OHD3_[BSA]_ = 12.2 + 3.3 × 25OHD2_[BSA]_; R^2^ = 0.91; p-value < 0.001; Fig. 2c). The 4.6% of the repeat testing (not exposed to BSA) sample that had non-zero 25OHD2 concentration were the values furthest below the contaminated 25OHD2 versus 25OHD3 regression line. Based on this finding, we identified the samples with expected non-zero 25OHD2 (this in-sample analysis correctly identified 98.7% of the true negatives and 66.7% of the true positives for non-zero 25OHD2).

### Correcting for the BSA-related 25OHD2

We returned to the full sample (n = 76,611) and plotted the contaminated 25OHD2 versus contaminated 25OHD3, and identified 4.6% (n = 3508) of the sample that was furthest below the line of regression between these two values (Fig. 2c). We assumed that these observations would have had non-zero 25OHD2 concentrations despite the BSA contamination. Within this set, we predicted their corrected 25OHD2 using a linear regression model based on repeat-test (contaminated 25OHD2 and 25OHD3) subset (corrected 25OHD2_[Corr]_ = 2.86 + 0.84 × 25OHD2_[BSA]_—0.23 × 25OHD3_[BSA]_; R^2^ = 0.81; p-value < 0.001). Apart from this subset, the remainder of the samples were recoded as 25OHD2 = 0 nmol/L.

### Correcting for the BSA-related 25OHD3

**Fig. 3:**
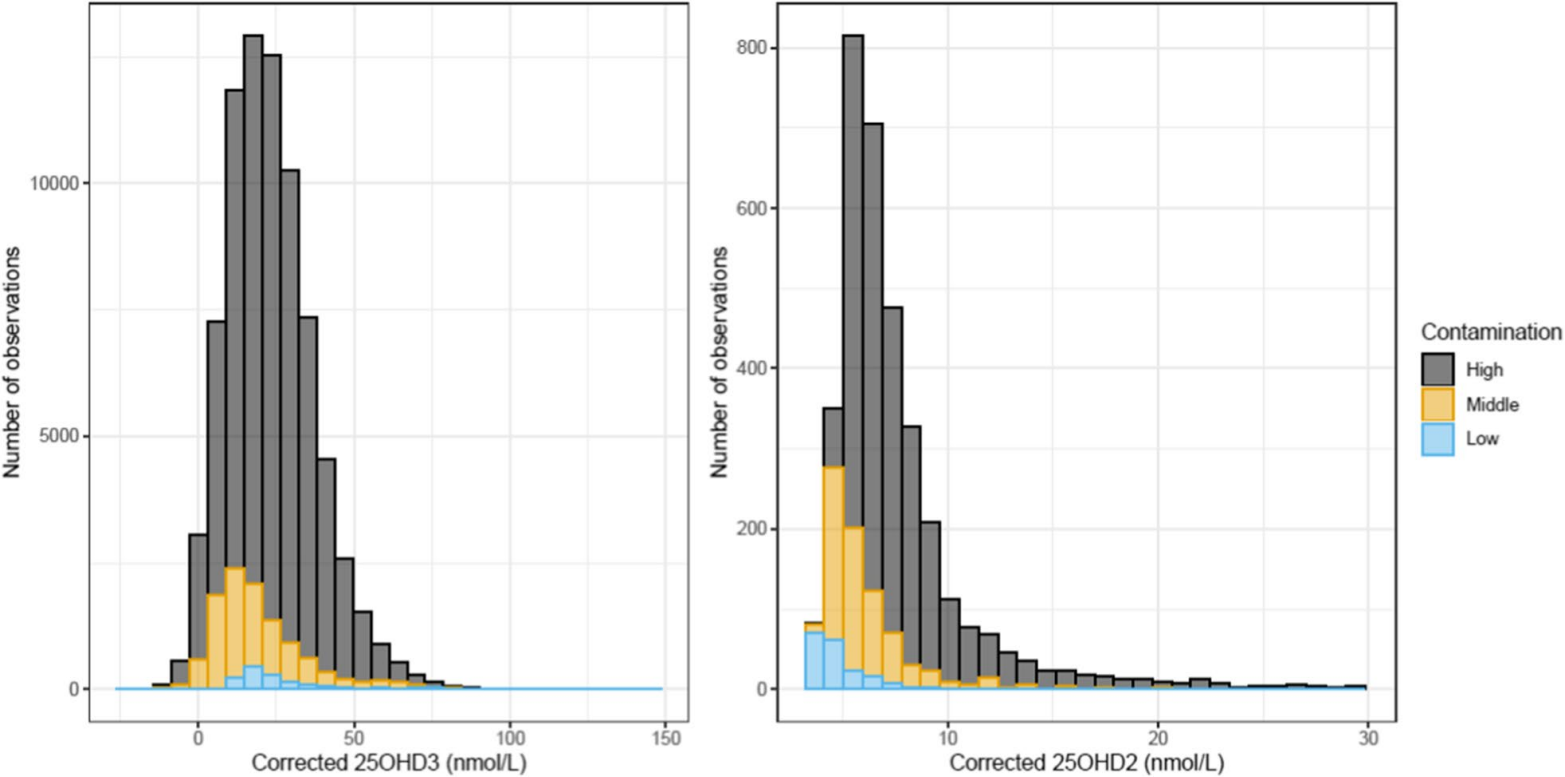
Histogram of corrected values of 25OHD3 and corrected values of 25OHD2 (among those with non-zero value) in nmol/L according to low-, middle-, and high-contamination of the samples (with average 25OHD2 levels per batch of less than 10 nmol/L, 10–20 nmol/L, or greater than 20 nmol/L, respectively)

Next, within the subsample with repeat testing, we plotted (a) the difference between contaminated minus uncontaminated 25OHD2, versus (b) the difference between contaminated minus uncontaminated 25OHD3. In light of our finding that 25OHD2 concentration could act as a proxy marker for BSA contamination, the difference scores were stratified into the low-, middle- and high- contamination subgroups. Based on the slopes and intercept of these difference scores (Additional file 1: Figure), we were then able to derive corrected values for 25OHD3 for the entire sample. The regression models were Diff_25OHD3_ = − 8.2 + 1.8* Diff_25OHD2_ for low contamination, Diff_25OHD3_ = 23.8 + 1.7* Diff_25OHD2_ for middle contamination, and Diff_25OHD3_ = 14.3 + 3.0* Diff_25OHD2_ for high contamination. The distributions for the corrected 25OHD2 and 25OHD3 for the three subgroups are shown in Fig. 3. Mindful that the two lower concentration subgroups only contributed 15% of the total sample, the corrected distributions were within expectations for dried blood spots based on samples previously analysed in our laboratory [1, 3, 19]. The values for 25OHD2 are all very low and the modes of these three subgroups are below the lowest level of quantification (less than 5 nmol/L) for similar assays based on dried blood spots [2, 12].

## Limitations

BSA could also contain other biologically interesting analytes which could bias results of future biomarker studies that have included BSA during sample preparation. The method outlined above was optimized for 25OHD2 and 25OHD3, it is not clear how BSA could impact on the precision of other assays. The use of BSA can influence the accurate measurement of vitamin D metabolites, however methods are available to use targeted repeat assays in order to develop models to correct the influence of this agent.

## Supplementary Information

**Additional file 1:** **Table S1.** Summary of the contribution of 25OHD2, 25OHD3 from BSA results. Mean of the concentration (nmol/L), standard deviation (±SD) and coefficient of variation (%CV) are calculated for each sample. None of the donors had detectable 25OHD2. **Figure S1.** Scatter plots of the difference (contaminated–uncontaminated) values of 25OHD3 and 25OHD2 in nmol/L according to low-, middle-, and high-contamination of the samples (with average 25OHD2 levels per batch of less than 10 nmol/L, 10-20 nmol/L, or greater than 20 nmol/L, respectively). These models based on the subsample with repeated measures were used to predict corrected values in the entire sample (R2 = 0.97; p-value < 0.001). The regression models were Diff25OHD3 = − 8.2 + 1.8* Diff25OHD2 for low contamination, Diff25OHD3 = 23.8 + 1.7* Diff25OHD2 for middle contamination, and Diff25OHD3 = 14.3 + 3.0* Diff25OHD2 for high contamination.

## Data Availability

Owing to the sensitive nature of these data, individual level data can be accessed only through secure servers where download of individual level information is prohibited. Each scientific project must be approved before initiation, and approval is granted to a specific Danish research institution. International researchers may gain data access through collaboration with a Danish research institution. More information about getting access to the iPSYCH data can be obtained at https://ipsych.au.dk/about-ipsych/.

## Abbreviations

BSA: Bovine serum albumin
25OHD2: 25-Hydroxyvitamin D_2_
25OHD3: 25-Hydroxyvitamin D_3_
