# Supplementary material for: A method to correct for the influence of bovine serum albumin-associated vitamin D metabolites in protein extracts from neonatal dried blood spots
Source: BMC Res Notes. 2022 Jun 3;15:194. doi: 10.1186/s13104-022-06077-1 (PMC9166528; doi:10.1186/s13104-022-06077-1)

**Additional file 1**

**Additional file 1 methods**

*The assessment of BSA samples*

In order to test the hypothesis that BSA samples contained exogenous 25OHD2 and 25OHD3, we added samples from 17 recently purchased batches of BSA to venous blood samples from the 11 individuals. For BSA batches where we found detectable 25OHD2 and 25OHD3, we then repeated the analyses in triplicate in order to compare the (a) mean and standard deviation, and intra-assay coefficient of variation of 25OHD2 and 25OHD3 in these individuals after the addition of BSA from the 17 samples, versus (b) the results from our standard assay (without BSA contamination).

*The assessment of 25OHD2 and 25OHD3 concentrations*

These samples were punched (2x3.2mm punch per well) in Nunc® MicroWellTM 96 well polystyrene plates. 130 µL extraction buffer (PBS containing 5 mL/L Tween20 and “Complete protease inhibitor cocktail with EDTA” – 1 tablet dissolved per 25 mL of extraction buffer) was added to each well. The plates were shaken one hour at 450 rpm at room temperature, after which 30 µL of each sample was transferred to a Thermo Scientific 96 well NUNC microtiter plate. 120 µL internal standard (reconstituted in acetonitrile and diluted to a working solution of 1:100 compared to the kit insert) was added. The plate was placed on an orbital shaker (450 rpm) for 10 min at room temperature. The precipitated proteins were removed by centrifugation for 30 min at 4000 rpm/3220 rcf (4°C). 80 µL of the supernatants were transferred into a 96 deep well plate already containing 400 µL ethyl acetate and 180 µL deionized water for the liquid-liquid extraction procedure. All samples were mixed well using the “pipetting-mixing” function on an electronic pipette (20cycles of pipetting 500 µL ups and down). Two phases were separated during a centrifugation at 700 rpm for 5 min (4°C). 200 µL of the upper organic phase (containing the purified vitamin D metabolites) was transferred to a Thermo ScientificTM WebSeal Plate+ 96-Well Glass-Coated Microplate. Samples were then dried down in an Eppendorf Bench Top Concentrator PlusTM (60°C). Vitamin D metabolites were derivatized with 20 µL of the commercial PTAD reagent (reconstituted in ethyl acetate and diluted to a working solution of 1:12). The plate was incubated on an orbital shaker (450 rpm) at room temperature for 30 min, after which the reaction was quenched by the addition of 50 µL ethanol. Samples were dried down in a concentrator and later reconstitution in 80 µL 1:1 acetonitrile/deionized water solution. The reconstitution was carried out by mixing the plate on an orbital shaker (450 rpm) for 10 min at room temperature. The plate was centrifuged at 3000 rpm/1811 rcf for 10 min at 4°C and transferred to the autosampler. Finally, 40 µL was injected into the LC-MS/MS system. The full details of the LC-MS/MS methods are provided elsewhere [1, 2]

For external quality control, the assay was validated according to standard reference material (SRM 972a and 972) from the National Institute of Standards and Technology (NIST). In addition, our laboratory participates in the Vitamin D External Quality Assessment Scheme (DEQAS)[3]. During the period when the iPSYCH samples were analyzed, our laboratory assessed 9 panels of 5 DEQAS standard reference samples (total samples n = 45). Across these samples, the percent bias from the target values ranged from -10.6% to 12.6%, which indicates acceptable precision.

References:

1. Keller A, Handel MN, Frederiksen P, Jacobsen R, Cohen AS, McGrath JJ, et al. Concentration of 25-hydroxyvitamin D from neonatal dried blood spots and the relation to gestational age, birth weight and Ponderal Index: the D-tect study. Br J Nutr. 2018;119(12):1416-23.
2. Nielsen NM, Munger KL, Koch-Henriksen N, Hougaard DM, Magyari M, Jorgensen KT, et al. Neonatal vitamin D status and risk of multiple sclerosis: A population-based case-control study. Neurology. 2017;88(1):44-51.
3. Carter GD, Berry J, Durazo-Arvizu R, Gunter E, Jones G, Jones J, et al. Hydroxyvitamin D assays: An historical perspective from DEQAS. J Steroid Biochem Mol Biol. 2018;177:30-5.

**Additional file 1 Limitations**

While the repeat test samples were based on a modest sample (n = 393), a larger sample would have been desirable. We will use the corrected values of 25OHD2 and 25OHD3 to predict subsequent risk of mental disorders. Because we are confident that the BSA contamination in the larger iPSYCH sample would have impacted on cases and controls equally (samples were batched according to date of birth, not cases status), this systematic bias in measurement accuracy of 25OHD2 and 25OHD3 would bias future epidemiological studies (e.g. the association between neonatal vitamin D status and later health outcomes) towards the null. Finally, we did not have access to samples of the original BSA (in order to assay for BSA-related 25OHD2 and 25OHD3). In addition, we did not have a record of which BSA batch was used for different samples, nor if there may have been errors in the amount of BSA added to the previous DNA extraction steps.

**Additional file 1: Table S1.** Summary of the contribution of 25OHD2, 25OHD3 from BSA results. Mean of the concentration (nmol/L), standard deviation (±SD) and coefficient of variation (%CV) are calculated for each sample. None of the donors had detectable 25OHD2.

| *Analytes* | *Native*  *Concentration (nmol/L)* | *BSA Lot# SLCD0987* | | |  | *BSA Lot#SLCC3894* | | |
| --- | --- | --- | --- | --- | --- | --- | --- | --- |
|  |  | *Mean (nmol/L)* | *±SD* | *%CV* |  | *Mean (nmol/L)* | *±SD* | *%CV* |
| 25OHD2 |  |  |  |  |  |  |  |  |
| Donor 1 | 0 | 14.8 | 1.3 | 9.2 |  | 7.7 | 0.4 | 6.3 |
| Donor 2 | 0 | 15.6 | 0.6 | 3.8 |  | 7.8 | 0.3 | 5.1 |
| Donor 3 | 0 | 15.9 | 0.3 | 1.6 |  | 7.3 | 0.3 | 4.6 |
| Donor 4 | 0 | 15.5 | 0.2 | 1.5 |  | 8.3 | 0.1 | 0.9 |
| Donor 5 | 0 | 15.2 | 0.3 | 2.1 |  | 8.0 | 0.1 | 1.8 |
| Donor 6 | 0 | 15.6 | 0.3 | 2.2 |  | 7.6 | 0.3 | 5.1 |
| Donor 7 | 0 | 14.8 | 0.7 | 4.7 |  | 7.0 | 0.2 | 3.3 |
| Donor 8 | 0 | 15.0 | 0.4 | 3.1 |  | 6.9 | 0.4 | 6.3 |
| Donor 9 | 0 | 13.9 | 0.9 | 6.5 |  | 6.7 | 0.3 | 5.7 |
| Donor 10 | 0 | 14.7 | 1.1 | 7.5 |  | 7.4 | 0.4 | 6.5 |
| Donor 11 | 0 | 14.3 | 0.3 | 1.4 |  | 7.1 | 0.8 | 11.6 |
| *Average contribution* | ***-*** | ***15.0*** | ***0.8*** | ***5.4*** |  | ***7.5*** | ***0.5*** | ***7.6*** |
|  |  |  |  |  |  |  |  |  |
| 25OHD3 |  |  |  |  |  |  |  |  |
| Donor 1 | 46.6 | 75.4 | 3.2 | 4.2 |  | 72.6 | 2.3 | 3.2 |
| Donor 2 | 25.8 | 52.6 | 1.9 | 3.7 |  | 47.3 | 1.1 | 2.5 |
| Donor 3 | 29.8 | 62.0 | 2.1 | 3.5 |  | 51.4 | 1.2 | 2.3 |
| Donor 4 | 27.9 | 52.3 | 0.8 | 1.6 |  | 46.8 | 1.5 | 3.2 |
| Donor 5 | 51.0 | 76.4 | 0.9 | 1.2 |  | 67.9 | 1.9 | 2.9 |
| Donor 6 | 59.4 | 98.9 | 10.4 | 10.5 |  | 81.8 | 0.6 | 0.7 |
| Donor 7 | 11.2 | 37.2 | 1.3 | 3.6 |  | 28.2 | 0.7 | 2.7 |
| Donor 8 | 47.8 | 74.5 | 1.0 | 1.3 |  | 72.3 | 1.5 | 2.1 |
| Donor 9 | 28.4 | 52.9 | 2.1 | 4.1 |  | 45.9 | 2.5 | 5.5 |
| Donor 10 | 32.3 | 54.4 | 1.2 | 2.2 |  | 51.8 | 0.7 | 1.3 |
| Donor 11 | 31.0 | 58.9 | 2.1 | 3.7 |  | 52.8 | 1.8 | 3.4 |
| *Average contribution* | ***-*** | ***27.7*** | ***3.0*** | ***11.0*** |  | ***20.9*** | ***3.0*** | ***14.6*** |

**Additional file 1: Figure S1.** Scatter plots of the difference (contaminated – uncontaminated) values of 25OHD3 and 25OHD2 in nmol/L according to low-, middle-, and high-contamination of the samples (with average 25OHD2 levels per batch of less than 10 nmol/L, 10-20 nmol/L, or greater than 20 nmol/L, respectively). These models based on the subsample with repeated measures were used to predict corrected values in the entire sample (R^2^ = 0.97; p-value < 0.001). The regression models were Diff_25OHD3_ = -8.2 + 1.8* Diff_25OHD2_ for low contamination, Diff_25OHD3_ = 23.8 + 1.7* Diff_25OHD2_ for middle contamination, and Diff_25OHD3_ = 14.3 + 3.0* Diff_25OHD2_ for high contamination.


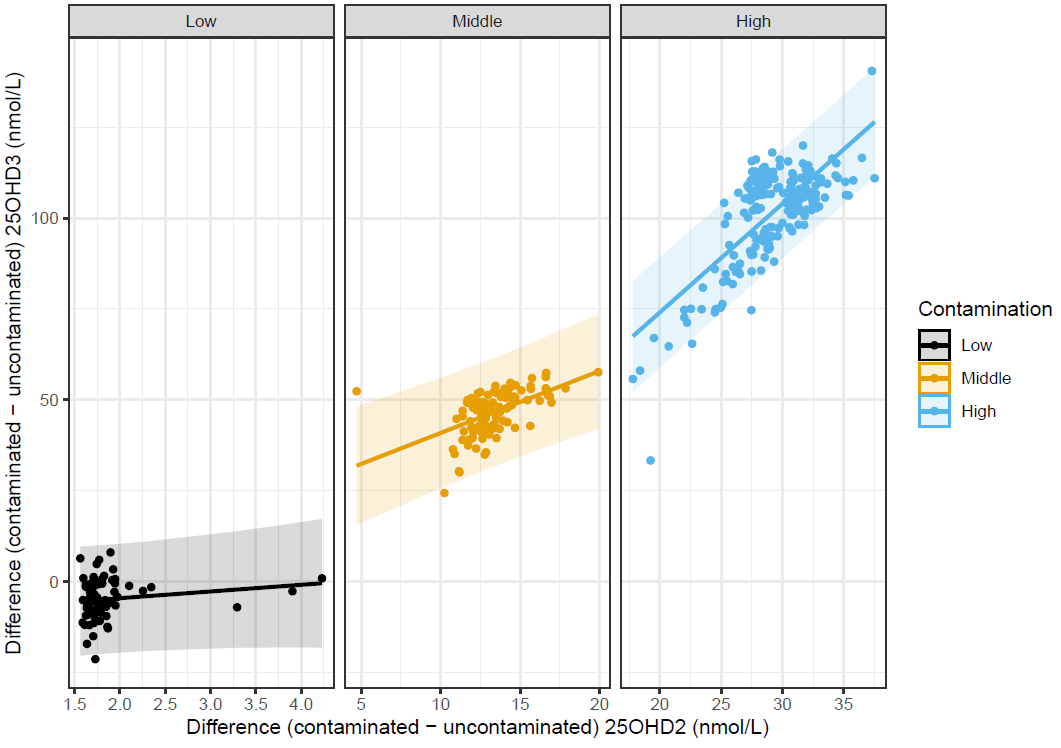

Supplement: Supplementary file 1 — Additional file 1: Table S1. Summary of the contribution of 25OHD2, 25OHD3 from BSA results. Mean of the concentration (nmol/L), standard deviation (±SD) and coefficient of variation (%CV) are calculated for each sample. None of the donors had detectable 25OHD2. Figure S1. Scatter plots of the difference (contaminated–uncontaminated) values of 25OHD3 and 25OHD2 in nmol/L according to low-, middle-, and high-contamination of the samples (with average 25OHD2 levels per batch of less than 10 nmol/L, 10-20 nmol/L, or greater than 20 nmol/L, respectively). These models based on the subsample with repeated measures were used to predict corrected values in the entire sample (R2 = 0.97; p-value < 0.001). The regression models were Diff25OHD3 = − 8.2 + 1.8* Diff25OHD2 for low contamination, Diff25OHD3 = 23.8 + 1.7* Diff25OHD2 for middle contamination, and Diff25OHD3 = 14.3 + 3.0* Diff25OHD2 for high contamination. [file 13104_2022_6077_MOESM1_ESM.docx]
